# The Medical Works of Paulus Ægineta, the Greek Physician, Translated into English; with a Copious Commentary, Containing a Comprehensive View of the Knowledge Possessed by the Greeks, Romans, and Arabians, on All Subjects Connected with Medicine and Surgery

**Published:** 1834-04-01

**Authors:** 


*The Medical Works of* PAULUS ÆGINETA, *the Greek Physician, translated into English; with a copious Commentary, containing a comprehensive View of the Knowledge possessed by the Greeks, Romans, and Arabians, on all Subjects connected with Medicine and Surgery.* By FRANCIS ADAMS, Esq., Surgeon, Author of "Hermes Philologus," &c. Vol. I.—London, 1834.

THERE has been great diversity of opinion, even among judicious men, on the advantages of studying the ancient writers on medicine; some maintaining that almost all that is valuable in the science is to be found in their works, and others contending that nothing but antiquarian enthusiasm can discover in them anything truly useful, or applicable to the present advanced state of knowledge. If the question were argued on the ground of immediate utility, truth would probably incline to the side of the latter, since there is no doubt that a man who has never read a word of Hippocrates or Galen, may nevertheless be a scientific physician, and an excellent practitioner; but we do not think this is the proper way of viewing the subject. It must indeed be admitted, that we know nearly all that the ancients knew, that we have corrected many of their errors, and greatly augmented the stock of knowledge which they bequeathed to us; but is there no benefit to be derived from tracing knowledge back to its sources? none from contemplating ideas which are now familiar, in all the simplicity and vigour with which they sprang from minds comparatively unwarped by prejudice, unbiassed by controversy, and conversant chiefly with the observation of nature? Is it unprofitable to learn how others have arrived at truth, or become entangled in error? In a word, is not the history of every subject essential to its true philosophy?

There is, moreover, much pleasure to a liberal mind in the extended survey of a science to which some of the highest of human intellects have in all times been devoted, and in marking the gradual progress of the fabric they have reared. If it be objected, that this pleasure is of an imaginative cast, it may be answered, that an object is never so ardently and successfully pursued as when the imagination illumines, without misleading, the course of the severer faculties.

But we must proceed to the immediate object of this article,—the work of Mr. Adams, a work of great labour and research, and by far the most learned that has been published in this country on the subject of ancient medicine, since Friend's History of Physick. If this were merely a translation of Paulus Ægineta, we should set our face against it, whether it were well or ill executed. To translate a classical author is at best a species of moral flaying,—it is to denude him of his natural tegument of language; and all English versions of the Greek or Latin physicians are especially to be reprobated, as tending to perpetuate that ignorance of the classical tongues which is already the disgrace of our profession. Mr. Adams' translation, however, is to be exempted from this censure, since it is merely intended as the basis of a very correct and comprehensive view of the theory and practice of ancient medicine; and he has selected his author judiciously, as the writings of Paulus afford a sort of compendium of Grecian medicine at its latest and most advanced period.

Such a work has hitherto been a great desideratum in medical literature, though its magnitude might well deter any one from attempting it. The labour is, however, more practicable than might at first be supposed, since, with reference to the authors who succeeded Galen, it consists only in pointing out the comparatively few particulars in which they differed from him. That extraordinary man, devoting to medicine all the resources of a vast intellect, a subtle though false philosophy, and a varied erudition, brought the study to the greatest perfection it was then capable of attaining; and, in the ensuing absence of all equal talent, his genius shone through the long lapse of fifteen centuries,

> Ἐν ἁμέρᾳ φαεινὸν ἄστρον
> Ερήμας δ' αἰθέρος; nor is there any other profane writer, except Aristotle, whose opinions have exerted a more permanent influence on the minds of men.

Such a work as Mr. Adams's is still an immense undertaking, for which, however, the great extent of his reading seems eminently to qualify him: he professes, indeed, "to have read every word of every ancient writer that has come down to us;" a bold assertion, but one which the perusal of his book affords no reason to call in question.

Four Greek physicians, after Galen, have been admitted into the number of the medical classics. They succeeded each other in the following order, and, in all probability, nearly about the periods severally annexed to their names, on the authority of Dr. Friend:

| | |
|---|---|
| Oribasius . . . . | A.D. 360 |
| Aetius . . . . . | 500 |
| Alexander of Tralles . . | 560 |
| Paulus Ægineta . . | 640 |

These authors were all, more or less, copiers of Galen, though they also drew from Hippocrates and other ancient authorities; and each, especially Aetius and Paulus, have given many results of their own experience. The voluminous writings of Oribasius consist principally of an epitome or interpretation of those of Galen, whose meaning he has in some passages rendered clearer. Paulus, again, professes to be chiefly indebted to Oribasius, speaking with much modesty of his own individual contributions, which however were numerous and important, for his surgery is much more complete than that of any preceding author; he is the first who treats of the obstetric art, and he is more particular than his predecessors on the diseases of females.

In the following passage, at the conclusion of the preface, Mr. Adams has, we believe, condensed all that is known of the history and æra of Paulus.

"Before concluding these prefatory remarks, it will be naturally expected that I should say something of the author whose work I have bestowed so much pains in translating and commenting upon. Here, however, I must regret that the information which I have to supply is exceedingly scanty and unsatisfactory. So little is known of him that it is not even ascertained in what century he flourished. Vossius is wholly undecided; Moreau and Le Clerc place him in the fourth century; Vander Linden and Convinguis, in the fifth; but Friend, Albertus Fabricius, Hutcheson, Sprengel, and most of the late writers of the ancient history of medicine, bring him down as low as the seventh century, upon the authority of Abulfaragius; an author, however, who on many occasions betrays such gross ignorance of chronology that no reliance ought to be put on any opinion of his on these matters. What confidence does a writer deserve who states, for example, that Andromachus, the physician who added the flesh of vipers to the celebrated electuary of Mithridates, lived in the time of Alexander the Great! that Dioscorides of Ain Zarba flourished in the reign of Ptolemy Physcon, namely, about 120 years before Christ, whereas it can scarcely admit of a doubt that the celebrated author of the Greek Materia Medica did not live earlier than the end of the first century of the Christian æra; and that Ruffus was cotemporary with Plato, when we have the authority of Suidas that he lived in the reign of Trajan! Dr. Milward, in his Epistle to Sir Hans Sloane, endeavours to settle the age of our author from the following train of inferences: In the first place, then, since Paulus quotes Trallian, and Trallian Aetius, it is quite certain that our author was posterior to both these writers. Now the age of Aetius may be made out from the following circumstances: Aetius mentions St. Cyril, archbishop of Alexandria, whose death is ascertained from ecclesiastical history to have happened as late as the middle of the fifth century. Nay, he also takes notice of a medicine much recommended by Petrus Archiater, chief physician of Theodoric, who was posterior to St. Cyril. We cannot possibly suppose it likely, then, that Aetius flourished earlier than the end of the fifth century. But what brings him still further down is the circumstance of his predecessor, Trallian, being mentioned by Agathias the historian, about the year 565. It would seem almost certain, therefore, that our author cannot have lived at an earlier period than the end of the sixth or the beginning of the seventh century. But, whatever may have been the period at which he lived, there can be no doubt that he attained great eminence in his profession, and continued to be looked up to as one of the highest authorities in medicine and surgery during a long succession of ages. His countryman Nonnus, although, if I recollect right, he does not mention him by name, gives a brief compendium of a considerable portion of his work; and Psellus does the same in Leonine verses.

"All the medical authors, in a word, of the distinguished Arabian period, quote his opinions in almost every page of their works, and never fail to recognise him as one of the most trustworthy of their Grecian masters. At the revival of literature in modern times, the Latin translations of the Arabians continued for a time to be the ordinary guides to practice; but, when the superior merit of their Greek originals came to be properly appreciated, our author rose again into high consideration. As a proof of this, I may mention that the surgery of Fabrice d'Aquapendente is made up almost entirely from his works. Portal therefore had no good occasion for representing him as "one of those unfortunate writers to whom posterity had not done justice." I admit indeed that for some time past, since professional research and the study of ancient models have been superseded by a restless desire of novelty in theory and in practice, he has not enjoyed that consideration to which he is justly entitled, but in this respect he has only shared the fate of other names equally eminent for their contributions to medical science, who have now been suffered to fall into neglect.

"Of the Latin translations of his works, the most celebrated is that of Cornarius, published by Henry Stephens, in his *Medicæ Artis Principes;* but which, after a careful examination, I have not found to be so trustworthy as I expected to find it. There once existed an Arabic edition by Honain, a Syrian physician, but of it I know nothing. The only part which has been translated into any modern language is the sixth book, a French translation of which was published at Lyons A.D. 1539. Of the original there are two editions, namely, the Aldine of 1528, and the Basle of 1538, neither of which is so accurate as could be wished."

The work of Paulus consists of seven books, of which the volume before us comprises the three first. Our remarks on this publication will be directed chiefly to the annotations of the editor, which constitute by far the most important part of it.

The first book treats of all that relates to Hygiene, of the influence of external agents on the system; of diet and regimen, as adapted to different ages, sexes, constitutions, and seasons; and, in the consideration of the *temperaments*, has afforded scope to Mr. Adams for an excellent outline of ancient physiology. In his annotations on the 63d and following chapters he comments

" *On the Characters of the Temperaments of the Brain.* The ancients divided the powers or faculties of the human body into the natural, the vital, and the animal. The brain they held to be the seat of the animal powers, that is to say, they considered it to be the organ from which sensation and motion are derived, and these, they maintained, are the powers by which animals are distinguished from vegetables. This doctrine is fully explained by Galen, in his work De Facultatibus Naturalibus, and by several of the Arabian authors, among whom I will venture to mention Haly Abbas as being particularly worthy of being consulted on this subject. The brain, then, was accounted the seat of the five external senses, and of muscular motion, which also was reckoned as one of the senses by Hippocrates, (De Insomniis, c. 1.) I may mention, by-the-way, that the late Dr. Brown, and the present Dr. Abercrombie of Edinburgh, adopt the arrangement of Hippocrates. Galen and his followers decidedly taught that the nerves of the senses are distinct from those which impart the power of motion, that the former derive their origin from the anterior part of the brain or cerebrum, and the latter from the posterior, called by the Greeks encephalis, (under this term they comprehended the cerebellum, tuber annulare, and medulla oblongata of modern anatomists,) or, from its process, the spinal cord. They maintained that the nerves of the finer senses are formed of matter too soft to be the vehicles of muscular motion; whereas, on the other hand, the nerves of motion are too hard to be susceptible of fine sensibility, Willis, and lately Mr. Charles Bell, in his reply to an article of mine, in the Medical Gazette, (May 2, 1829,) on the opinions of the ancient physiologists with regard to the nerves, deny that the nerves of sensation are softer than those of motion. Ackerman and Malacarne, however, maintain that the opinion of Galen on this point is perfectly correct, and with them I entirely agree. Let any person attentively examine the gustatory and muscular nerves of the tongue, and he will be sensible of the superior softness of the former. As my limits would not admit of my giving a full explanation of this celebrated theory, which was lately revived with great eclat, I must be content with referring to Galen, de *Usu Partium*, lib. ix. *de Administ. Anat.* lib. vij.; to Haly Abbas, *Theoricæ*, lib. iv.; to Averrhoes, *Colliget*, iij. 33; to Avenroar, ij. 7; and to Rhases, *Continens*, lib. i.

"The ancients were also of opinion, that the brain is the coldest viscus in the animal frame, being in this respect the antagonist of the heart, the heat of which they supposed that it counteracts: see Aristot. *De Part. Anim.* ij. 7, and Pliny, H. N. xi. 49. There appears to be some foundation for this opinion, since, as is remarked by Haly Abbas, those parts of the body which are vascular, and contain much blood, are naturally hot; whereas such as contain little blood are comparatively cold. Of this latter class are the brain, nerves, and fat. *Theor.* lib. i."

In this passage we may discover traces of the only evil result which is wont to follow an enthusiastic addiction to the literature of the ancients, namely, a disposition to attribute to them opinions and discoveries which are entirely of modern origin. Thus Mr. Adams evidently wants to make out that the theory of the *muscular sense* originated with Hippocrates. The passage he refers to is the following:

ἡ γὰρ ψυχὴ ἐγρήγορεν. ὅταν μὲν οὖν σώματι ὑπηρετοῦσα ᾖ, ἐπὶ πολλὰ μεριζομένη, οὐ γίγνεται αὐτὴ ἑωϋτῆς, ἀλλ' ἀποδίδωσί τι μέρος ἑκάστῳ τοῦ σώματος, ἤγουν τοῖσιν αισθητηρίοισιν. ἀκοῇ. ὄψει. ψαύσει. ὁδοιπορίῃ. πρήξει. καὶ πασῃ, τῇ του σώματος διανοίῃ.

Now Hippocrates cannot surely call ὁδοιπορίη (*walking* or *running*,) a sense, which would be a solecism in terms: he merely enumerates it along with the senses, as dependent on the action of the mind. So far indeed was the Father of Medicine from having any theory connected with muscular action, that he had no conception of muscular action at all; for he attributed the movements of the body and limbs to the tendons, and believed that the use of the muscles was merely to retain the different parts of the frame in connexion with each other. This is clear from the following passage:

τὰ ὀστέα τῳ σώματι στάσιν καὶ ὀρθότητα, καὶ εἶδος παρεχονται. τὰ δὲ νεῦρα, κάμψιν, καὶ ξύντασιν, καὶ ἔκτασιν. αἱ δὲ σάρκες καὶ τὸ δερμα, παντων ξύνδεσιν, καὶ ξυνταξιν.

(*De Ossium Natura, ed. Foes*, p. 277.)

With equal futility Mr. Adams endeavours to give to Erasistratus, Aretæus, or Galen, all the merit of the beautiful modern discovery of the distinct functions of the anterior and posterior columns of the spinal marrow. In his note on apoplexy and paralysis, in the third book, he makes some more explicit remarks on this subject:

"It is impossible to admire too much the brief but comprehensive account of apoplexy and paralysis given by Aretæus. He states decidedly that there is sometimes a loss of motion alone, and sometimes of sensibility; the reason of which he supposes to be that the sensatory and motory nerves are distinct from one another. This is the germ of a theory fully expanded afterwards by Galen, and lately revived by Sir Charles Bell, of London, as a new discovery. It appears, indeed, from the anatomical works of Ruffus, that the famous Erasistratus had attempted a similar classification of the nerves. Galen, however, has the merit of fully establishing the truth of the theory; and all subsequent writers on physiology stated it nearly in the same terms that he does, until ancient authority in medicine and its cognate sciences came to be despised, when it was entirely overlooked, until, as we have already mentioned, it was revived by Sir C. Bell."

As the writings of Galen are too voluminous for easy reference, and too expensive to be generally accessible, the following sketch of his more prominent views on the subject of the nervous system may not be unacceptable to the reader.

The nervous system consists of the brain, the spinal marrow, and the nerves.

The brain, including the cerebrum and cerebellum, is the immediate seat of the mind, and, as such, the primary organ of sensation and motion.

The brain is composed of the same substance as the nerves; the anterior portion is the softer, and gives origin to the nerves of sense; the posterior is harder, and gives origin to nerves of motion.

The spinal marrow is a production from the cerebellum, which, however, it exceeds in consistence. It contains just so much nervous matter as is necessary to form about sixty pair of nerves, which are distributed to each part according to its demands for nervous energy.

Nerves have three uses: to communicate to the organs of sense their respective sentient faculties; to excite motion in the organs of motion; and to enable the organs of the body in general to discern what might be injurious to them. *(De Usu Part.* lib. v. c. 9.)

The nerves of the senses are soft, like the part of the brain from which they are derived; they all rise from the anterior part, and pursue a straight course to the organs which they supply.

The motory nerves are hard, arising from the posterior part of the brain, and the whole of the spinal marrow: these pursue a circuitous course to the parts to which they are distributed.

Although Galen thus recognised a distinction between the sensatory and motory nerves, he conceived that this difference of function arose merely from difference of *consistence*. His idea appears to have been a mechanical one,—that the soft nerves were more susceptible of impressions, and the hard nerves less impressible, but stronger, and therefore better fitted for action. He had not the smallest notion of any original difference in the nature of the nervous power communicated by the two classes of nerves, nor that the parts of the central mass from which they spring were respectively the depositories of a sensific and a motific principle, distinct in their nature, and dependent on different modifications of the vital power. That he was entirely ignorant of all this, is evident from his maintaining that the hard motory nerves, although comparatively insusceptible of sensation, do nevertheless possess that faculty in a subordinate degree, and sufficiently to produce the general sense of *touch:* and also that a nerve which originates as one of *sensation* from the soft part of the brain, may, at some part of its course, become condensed in its texture, and assume the office of a *motory* nerve. (*De Usu Partium*, c. xiv.)

The brain, according to Galen, gives origin to seven pair of nerves.

1. The *optic*, the largest and softest of the cerebral nerves, rise from the thalami optici, and joining each other, but without decussation, again separate, and perforate the ball of each eye, where they are ultimately distributed to the crystalline lens, then supposed to be the more immediate organ of vision.

2. The second pair (the third of the modern arrangement,) are smaller, but harder than the first: they rise toward the back part of the brain, and pass to the muscles of the eyeball, to which they communicate motion.

3. The third pair (fifth) are very hard; they rise towards the base of the brain, at the junction of the cerebrum and cerebellum. Only two of the branches of this nerve were known to Galen, viz. the superior and inferior maxillary: these are correctly stated to be distributed to the tongue, where they form the organ of *taste;* to the muscles of the upper jaw, and those of the face; to the gums, and roots of the teeth.

4. The fourth pair (apparently the first branch of the fifth,) are small, but still harder than the preceding: they rise from the base of the cerebellum, and pass through the same foramina as the third pair. They go to the palate, where they also minister to the sense of taste.

5. The fifth pair, or *auditory*, are also very hard; they rise a little behind the last, and go to the internal ear.

6. The sixth pair *(par vagum)* are still harder, and rise farther back. They supply many branches to the gullet, stomach, and viscera, and each sends back a *recurrent* nerve to the muscles of the larynx.

7. The seventh pair *(glosso-pharyngeal)* are the hardest of all the nerves of the encephalon, and rise from the junction of the spinal marrow with the cerebellum; they accompany the sixth pair for a short distance, and then, separating from them, are distributed to the tongue, which they endow with motion, and to the muscles of the larynx.

Galen was unacquainted with the olfactory nerves, though he describes the bulbs from which they are given off: he believed that the extremities of the anterior ventricles of the brain formed the organ of smell.

The ganglionic system of the great sympathetic nerve was unknown to him.

Such is a slight outline of the nervous system according to Galen. We have avoided the minutiæ, because they are obscure, and for the most part inaccurate.

The reader will perceive how little reason there is for Mr. Adams's assertion, that Galen anticipated Sir Charles Bell's discovery of the sensatory and motory nerves, which consisted not in suggesting the probability that some nerves were destined for sensation, and others for motion, but in shewing, with reference to the spinal cord, that the anterior columns have exclusively the power of communicating motion, and the posterior that of receiving the impressions of sense. The diversity of function in the sensatory and motory nerves was ascribed by Galen to difference of texture, by Bell to difference of vital property; and, with regard to the respective origin of these two classes of nerves, Galen *conjectured*, and was wrong, Bell *demonstrated*, and was right.

Truly Sir Charles and M. Magendie will be sorely aghast; for, after these gentlemen have been contending for some years about the prior right to this discovery, and we in our simplicity have conceived the question to be settled in favour of the former, in walks Galen, ushered by Mr. Adams, and plays the part of the umpire in the fable of the disputed oyster!

Jesting apart, Galen knew about as much of this matter as

Goliah; and we cannot help observing, that modern erudition may be better employed than in attempting to rob contemporary merit of its just honours; and that it is a poor compliment to the ancients to dress them in borrowed robes, when they look so majestic in their own.

"*On the Characters of the Temperaments of the Stomach.* We shall now state briefly the opinions of the ancients with regard to the functional office of the stomach. Actuarius says, 'I am of opinion that there are four species of concoction, which are performed in different parts of the body: the first in the stomach; the second in the vena ramalis (vena portæ?) meseraic veins, and concave part of the liver; the third, in the convex part of the liver and veins proceeding from it; and the fourth, consisting of fabrication or assimilation which takes place in the extreme parts of the body.' *De Urinis.* The various modes of change or concoction which the food undergoes in the body are minutely described by Macrobius, Saturnal. lib. vij. In another place Actuarius says, 'digestion is performed by moderate heat and moisture.' *De Spiritu Animali,* p. ii. § i. Alsaharavius in like manner states, that the digestive faculty depends partly on the heat, and partly on the humidity of the stomach. Bract. tr. xvj. c. i. It is impossible not to see that the gastric juice is alluded to in these passages. It is particularly stated of Asclepiades, that he held digestion to be the solution of the food. See C. Aurelianus, i. 13. And that the ancients were aware that the stomach secretes a fluid possessed of solvent properties, is put beyond a doubt, by the following extract from the works of Haly Abbas. Speaking of the changes which the food undergoes in the mouth and stomach, he says: 'Immutantur cibi in ore, retinenturque, et flegmati admiscetur quod digestum est, calorque ei datur. Quod autem flegma hoc hujusmodi sit, signum nobis est, quod impetigines et sarpedones curat, quædam maturat ulcera, scorpiones necat. Hac ergo de causa et in ore cibus immutatur. Sic et stomachus ipsum immutat: ejus etenim circum amplectitur substantia, quasque habet imprimit qualitates, immutaturque ipsius naturali calore cibus: *sed et quoniam cibus ipse in eo flegmati admiscetur humido.*' *Theor.* iv. 3. The whole bearing of this passage, but more especially the last clause, puts it beyond a doubt that the process of digestion was supposed to be performed, in a certain measure, by the solvent powers of a fluid secreted in the stomach. And the ingenious Alexander Aphrodisieus, in like manner, treating of the digestion of mustard, pepper, and other acrid substances, says decidedly, that their acrimony is dissolved in the copious fluid of the stomach. *Probl.* i. 30: see also Macrobius, *Saturnalia,* lib. vij. 8. He calls it *ventralis humor.* Part of the process was, no doubt, supposed to be performed indirectly by heat; and deservedly, for even Spallanzani was compelled to admit that the comparative temperature of animals exerts a considerable influence on their digestive powers. Hence, as was stated by Averrhoes, and as is confirmed by Cuvier, birds, which are the warmest class of animals, likewise digest the fastest. At all events, the ancients were all aware that digestion is not a mechanical but a vital process, being performed by the principle of life. "Digestion," says Averrhoes, "is performed by concoction, and the concoction is influenced by heat, not that the first mover in the operation is heat, but the nutritive soul; because the operations of heat are indeterminate, and not directed to any manifest end."

"*Colliget.* v. 3. In the Averroeana, or Letters from Averrhoes to Metrodorus, the doctrine of a gastric menstruum is discussed with singular ability. Metrodorus states, that 'he found by the writings of the physicians and philosophers of these times, that they make the *menstruum*, as they call it, whereby both appetite is provoked, and the food in the stomach is digested, to be a certain juice or humour in the stomach,' &c. Averrhoes denies that this menstruum acts by its acidity alone."

Mr. Adams has here succeeded in shewing that the ancients made a near approach to the true theory of digestion: it does not appear, however, that they had any notion of the gastric fluid being a secretion from the vessels of the stomach itself. With respect to the precise manner in which this solvent acts on the food, they may fairly be said to have known as much as we do, that is—nothing.

"*On the Characters of the Lungs.* The ancients were of opinion, that the lungs are an accessory organ, made to administer to the heart. 'It is the heart,' says Aretæus, 'which imparts to the lungs the desire of drawing in cold air." They, of course, were aware that respiration is the functional office performed by the lungs; and, respecting the uses of this vital process, they were pretty much agreed. Aristotle, indeed, and the older physiologists, taught that refrigeration is the purpose of respiration; but Galen explains that, probably, they were at a loss for a proper term, and used it in the sense of ventilation. Galen, himself, perpetually inculcates, that by respiration the vital heat is regulated, being increased or diminished according to the circumstances of the animal. Another purpose, which he, Haly Abbas, and other ancient physiologists, supposed to be performed by respiration, is the evacuation of the fuliginous vapours of the blood. Galen was aware that respiration produces the same effect upon atmospherical air that combustion does, and that it is equally necessary to the one process and the other. See the treatises of Aristotle and Galen on Respiration, and Haly Abbas, *Theoricæ*, lib. iij. The following extract from Haly contains the summary of what we have been stating: 'Respiration is necessary for the sake of the heart, which is the fountain, and, as it were, the focus of vital heat, whence it is diffused over the rest of the body. It requires some aerial substance to ventilate the heat and ebullition of the heart, and in order to evacuate the fuliginous vapours which are found in it.'"

If Galen had been aware of the chemical constitution of the atmosphere, and the circulation of the blood, it is very probable that he would have anticipated Dr. Crawford in his theory of animal heat. No higher praise can be accorded to the philosopher of Pergamos, than by saying that his views, on almost every subject, were the best which the state of physical science at the time admitted.

Notwithstanding the confidence with which the modern chemical theory of animal heat has been embraced by some very distinguished physiologists, we are much mistaken if a few years will not find opinions on this subject as unsettled as ever.

"*On the Temperaments of the Heart.* In the ancient system of physiology, the heart was considered as the seat of the vital powers, its office being the preservation of the innate heat of the body. The philosopher Aristotle had pointed out the connexion between heat and vitality, and had taught that the heart, as being the centre of heat, is the prime organ in the animal frame. Hence, as his commentator, Averrhoes, remarks, it is the *primum movens et ultimum moriens.* Galen however maintained, with Hippocrates, that the animal frame is a circle, having neither beginning nor end, and that, consequently, it has no prime organ. He taught that the brain does not, properly speaking, derive its powers from the heart, nor the heart from the brain; but that these organs are mutually dependent upon one another, the heart being indebted to the brain for supplying the parts concerned in respiration with muscular energy, and the brain being indebted to the heart for its vital heat, without which it could not continue to be the vehicle of sensibility and motion. We have mentioned in the preceding chapter, that the ancient physiologists looked upon respiration as being a process similar to combustion. Agreeably to this idea, they compare the heart itself to a lamp, its vital heat to the flame of the lamp, and the blood to the oil which feeds the flame. See Galenus, *de Usu Respirationis*, Alexander Aphrodisieus, *Probl.* i. 16. The heart, then, was supposed to convey heat to all parts of the body, by means of the animal spirits incorporated with the blood in the arteries. Respecting the contents in the arteries, two hypotheses divided the ancient schools of medicine. The first was that of the celebrated Erasistratus, who maintained that the arteries do not contain a fluid, but merely certain airs or vapours. This hypothesis was revived about seventy years ago by Professor Rosa, in Italy; and lately it found a zealous abettor in my lamented friend, Mr. George Kerr, of Aberdeen. The other hypothesis was that of Galen, who keenly attacked this, as he did most of the tenets of Erasistratus, and endeavoured to prove, by experiment, observation, and reasoning, that the contents of the arteries is blood, mixed indeed with a certain proportion of heat and airs, but in every respect a fluid, little different from that which is contained in the veins. It was also part of his system, that the right cavity of the heart attracts blood from the liver, and conveys it to the left, from which it is diffused all over the body by the arteries. He taught that, at every systole of the arteries, a certain portion of their contents is discharged at their extremities, namely, by the exhalents and secretory vessels; and that at every diastole a corresponding supply is attracted from the heart. He decidedly inculcates that it is the expansion, or diastole, of the artery which occasions the influx of the blood, and not the influx of the blood which occasions the expansion of the artery. Though he demonstrated the anastomosis of arteries and veins, he nowhere hints his belief that the contents of the former pass into the latter, to be conveyed back to the heart, and from it to be again diffused over the body. Of the greater circulation of Harvey he certainly had no idea. In a word, his system appears to have been nearly, or altogether, the same as that afterwards taught by the unfortunate Servetus.

"In proof of the opinions which I have attributed to Galen, I refer the reader to *An Natura Sanguinis sit in Arteriis, Administ. Anatom.* vii. 15; *de Usu Partium*, lib. vi. and lib. vii., 7, 8, 9; *de Placitis Hippocr. et Plat.* i. 5. See also Averhhoes, *Collegit.* ii. 8, 11, 9, *Collect.* § 1, 9; *In Cant. Avic.* tr. i. p. 1; Avicenna, lib. iii. fen. xi. tr. 1; Actuarius, *de Spiritu Animali*, p. 1, § 6, *de Causis Urinarum*, ii. 2; Nensesius, *de Natura Hominis*, § 24.

"With regard to the passages collected by the ingenious M. Dutens, from the works of Hippocrates, Plato, Nensesius, Pollux, and Theodoret, to prove that the ancients were acquainted with the circulation of the blood as taught by Harvey, I shall only remark that, after having attentively considered them, I cannot but draw the conclusion that some of these authors must have had, at least, an obscure idea of this doctrine, although in general these passages may be understood to refer merely to the lesser circulation, and the movement of the blood from the centre to the extremities, as maintained by Galen. See Dutens, *Origine des Découvertes attribuées aux Modernes*, p. 157; also Drelincurtius, *de Lienosis Epimetris.*"

All this is very fairly and candidly stated. We trembled lest it should turn out that the circulation had been discovered by Galen or Hippocrates, or perchance by Æsculapius or Hermes Trismegistus; but we are happy to find that Mr. Adams, though evidently like ourselves a "laudator temporis acti," is nevertheless disposed to concede a few things to the moderns, and is not one of those who believe that the latter half of the world's history is superfluous, and a mere lame imitation of what went before.

"*On the Temperaments of the Liver.* According to the views of the ancient physiologists, the liver is the seat of the natural powers, being the grand organ of sanguification, and the blood being the pabulum which nourishes the whole body. That the liver performs an important part in the fabrication of the blood, seems probable from all the veins of the stomach and upper portion of the intestines passing to the liver, whereby it is to be supposed that a considerable proportion of the nutritive juices will be conveyed to it, and from this viscus being proportionally large in the fœtus when it is much required to form blood, and cannot be supposed necessary for any other purpose. In fact, the late experiments of professors Tiedeman and Gmelin seem to prove that the liver is concerned in carrying off the recrementitious part of the blood, or, to use the language of modern chemistry, in decarbonising it. *Recherches Exper. sur la Digestion.* The ancients taught, that the liver, by its attractive power, attracts the chyle from the stomach; that, by its retentive, it retains the same until the alterative convert it into blood; and then the expulsive separates the superfluities of the blood, namely, the bile, and conveys them to the gall-bladder. See Galenus, tom. ij. p. 285. *Ed. Basil.* And Avicenna, lib. iij. fen. 4, tr. 1.

"Aristotle held that the spleen is part of the hepatic system, *de Partibus Animal.* iij. 7. His commentator, Averrhoes, in like manner, considers the spleen as a second liver. *Collect.* i. 9. Their reasoning, on this point, appears to me exceedingly acute and conclusive."

The second book of Paulus treats at length of febrile affections. The graphic descriptions of pestilential fevers by several ancient historians and physicians are well known, and the *causus* of Hippocrates, so finely illustrated by Aretæus, has been a frequent theme with the medical antiquary. Passing over these, therefore, we extract the remarks of Paulus, on the

"*Diagnosis and Cure of Fevers with an Erysipelatous Affection.* Fevers accompanied with an erysipelatous affection about the viscera, may be known by the vehement effervescence and strong pain in the part, also by the thirst and inordinate burning; in a word, by the symptoms of bitter bile putrefying along with a deficient blood. They are to be treated in this manner: the patient must abstain altogether from the bath, and at the acme of the complaint, use the very coldest water. But it must not be used at the commencement, but cold things are to be applied externally; and if this is not sufficient, they must be taken internally. Lettuces and such like things are particularly befitting. The juice of the lettuce is likewise a seasonable application externally, also that of the house-leek (*semper-vivum,*) and such like cooling things. We may use the following application, which is an excellent one: squeezing out the juice of some cooling thing, we put it into a mortar with purslain, then pound and strain it; at the time of using, we mix a little polenta with it, and place it in cold water to cool it. A piece of cloth folded double is to be put into it, and afterwards applied to the hypochondrium, and not suffered to remain, but another cold one must be substituted. We sometimes mix the oil of unripe olives."

On this subject Mr. Adams adds the following observations:

"This chapter is mostly taken from Oribasius, *Synops.* vj. 20. On erysipelas of the lungs consult Hippocrates, *de Morbis*, i. 13, and ij. 53. The following are the symptoms of fever connected with erysipelatous inflammation, as detailed by Alexander. The patient experiences thirst more strikingly than in any other case, throws the clothes off his body, has exacerbations every third day, with bilious and ichorous discharges from the bowels; those in whom erysipelatous inflammation is seated in the lungs have not so intense thirst, but breathe thick and large, their cheeks are red, tongue rough, they are delirious, and long rather for cool air, and are more benefited by it than cold drink, which ought rather to be given to those who have erysipelatous inflammation in any other part, whereas those in whom the lungs are affected ought to be supplied with cool air. In ordinary cases, he approves of giving cold water to extinguish the fever, but says that he has seen patients brought to imminent danger by the unseasonable application of cold cataplasms and clysters. Aetius states that fevers are kindled by the parts about the bowels, liver, and lungs, being attacked with erysipelas. Like our author, he approves of cold drink, cool air, and cold applications to the part affected. The acute affection of the vena cava, which is minutely described by Aretæus, ought probably to be ranked with the diseases which we are now treating of. *De Morb. Acut.* iij. 8. He recommends for it venesection and the refrigerant plan of treatment. *Cur. Morb. Acut.* ij. 7. We have stated in the preceding chapter that Palladius refers one variety of ardent fever to erysipelas of the lungs. A similar account of these affections is given by Avicenna, lib. iv. fen. 1, tr. 4, c. 13, 14, 15; and by Rhases, *ad Mansor.* x. 15, *alibique.*

"I can draw no information from modern works to illustrate the opinions of the ancients respecting the febrile affections treated of in this chapter. It does not seem to be suspected now that erysipelas ever attacks the lungs or bowels; and yet, as this disease when it occurs externally is known to be seated principally in the epidemics, and as the epithelium, or membrane which lines the internal cavities, is admitted to be a prolongation of it, (see Kaan Boerhaave, *Perspiratio Dicta Hippocratis*,) it would seem probable *a priori* that the diseases of both portions of it should be alike. That fevers are often complicated with ardent affections of the lungs and bowels, and bilious appearances, we all admit; and it might be worth while to inquire whether such diseases be of an erysipelatous nature."

The ancient writers appear to have used the term erysipelas in a very vague acceptation, as might indeed be expected, from their want of all defined ideas on inflammation in general. Hippocrates nowhere exactly defines erysipelas, and his descriptions of this affection, as occurring in particular parts and organs, are too heterogeneous to afford any data for a general definition. Under the name of erysipelas of the lungs, Dr. Huxham thinks that Hippocrates described "inflammations of the mediastinum, pericardium, and membranes of the lungs." (*Dissertation on Pleurisies and Peripneumonies*, c. iv.) The conjecture is probable.

We quote the description given by Hippocrates of erysipelas of the parts about the throat; on comparing which with the treatises of Fothergill and Huxham on the Putrid Sore-throat, it is difficult to avoid believing that this severe disorder, or one very like it, was well known to the Father of Medicine.

"Exulcerations of the fauces attended with fever are of serious portent; but if they be attended with any of the symptoms which we have before pointed out as bad, the patient is to be declared in danger. That form of cynanche is the severest and most speedily fatal, which presents no peculiar appearance either in the fauces or on the neck, but is accompanied with great pain and orthopnœa; it proves fatal, by suffocation, on the first, second, third, or fourth day of the attack. That form of the disease which is attended with similar pain, but also with swelling and redness of the fauces, is very fatal, but longer in its course than the last mentioned, if the redness be vivid. Of still longer duration is the case in which the redness appears not only in the fauces, but on the neck, and patients thus affected generally recover, if the erysipelas continues on the neck and chest, and does not return inwards."—(*Lib. Prænot. ed. Foesii*, p. 45.)

Hippocrates describes what he calls erysipelas of the uterus, in the book *de Natura Muliebri, ed. Foes*, p. 567, and that *de Morb. Mulier.* p. 663, in both which places, as also in the *Aphorisms*, lib. v. 43, he states that it is always fatal when it occurs in a pregnant woman. These descriptions are to us unintelligible; we have no idea what disease is meant to be delineated.

Galen has described erysipelas accurately as it appears on the surface, and inculcates its connexion with disorder of the biliary system, (*ad Glauc.* lib. ii.) On the whole, it is probable that the fevers connected with erysipelas, here mentioned by Paulus, were nothing more than those accompanied with common visceral inflammation. Mr. Adams is correct in stating that erysipelatous affections of internal parts have not been sufficiently attended to by modern writers. Frank alludes to this kind of inflammation in the membranes of the brain and spinal cord, but does not tell us by what peculiar symptoms it is indicated. (*De Curand. Hom. Morb.*, lib. ii. p. 51.) It is not improbable that the inflammatory affections of internal organs, accompanying fevers of an asthenic type, may sometimes partake of this nature. Dr. Douglas believes the worst form of puerperal fever to be connected with erysipelatous inflammation of the peritoneum; and Huxham, an author whose acute observation and sound judgment entitle all that he says to attention, states, when speaking of malignant fevers, that "an itching, smarting *red rash* commonly greatly relieves the sick; and so do the large, fretting watery bladders, which many times rise up on the back, breast, shoulders," &c. (*Essay on Fevers*, c. viii.) This relief may possibly arise from the metastasis of internal erysipelas to the surface.

The occasional extension of erysipelas of the head and face to the parts within the cranium, affords an opportunity of studying the symptoms of this form of inflammation when it attacks an important internal organ.

In an appendix to his commentary on the second book of Paulus, Mr. Adams introduces an interesting account of *smallpox* and *measles*, as described by the Arabian writers, maintaining correctly that it is to them we are indebted for the earliest notice of these diseases. We regret that our limits will not allow us to enter into this subject. We may remark, in passing, that Avicenna affirms that smallpox may occur twice in the same individual. "Et multoties quidem variolatur homo duabus vicibus, quando aggregatur materia ex expellendum bis." (*Canon.*, lib. iv. tr. 4, c. 6.)

Measles (*morbilli*) were confounded with smallpox even as late as the time of Sydenham. We do not know when the vernacular term *measles* began to be applied to this disease: we learn, however, from old John of Gadesden, who wrote early in the fourteenth century, that the term was in use in his day, but applied to a different affection: "Sicut autem punctilli duplices magni et parvi; de parvis dictum est. Magni sunt infectiones latæ, rubræ, et obscuræ, apparentes in tibiis pauperum, et consumptorum, ad ignem sine calceis continue fere sedentium; et vocatur Anglicè *mesles*." (*Rosa Anglica*, p. 1041, ed. Schopfii, 1595.)

The third book of Paulus is tolerably comprehensive, treating "of topical complaints, beginning with the head and ending with the toes."

The chapter on *headach* is worthy of attention, as presenting one of the few instances in which the ancients attributed diseases of distant organs to sympathy with the stomach; a doctrine which has communicated a new aspect to the medical theory and practice of the present day.

Among the remedies for this complaint we may mention one recommended by Scribonius Largus, which is absurd enough in itself, but is remarkable as the first instance on record of the application of *galvanism* to medicine. "Capitis dolorem quamvis veterem et intolerabilem, protinus tollit, et in perpetuum remediat *torpedo viva nigra,* imposita eo loco qui in dolore est, donec desinat dolor, et obstupescat ea pars: quod quum primum senserit, removeatur remedium, ne sensus auferatur ejus partis, &c. (*De Composit. Medicament.* c. 1.) This prescription, like many others of Scribonius, has been copied nearly verbatim by that most impudent of plagiaries, Marcellus Empiricus. Scribonius seems to have been very partial to black torpedos: he recommends their application to the feet in gout. (*Op. citat.* c. 41.)

Mr. Adams has a long and interesting note on *the diseases of the eye.* He remarks, however, with his usual partiality, "One may venture to affirm that, whoever will carefully study the works of all the ancient authors referred to above, will find every subject connected with diseases of the eye treated of so fully and judiciously, that he will not stand much in need of consulting modern writers for additional information."—Really this is too much!

"Quodcunque ostendis mihi sic, incredulus odi."

If there be a department of the healing art in which real knowledge is almost exclusively of modern acquisition, it is ophthalmic surgery.

We applaud Mr. Adams's endeavours to familiarise the writings of the ancients, whom none can hold in higher estimation than ourselves; but any attempt to set them up as guides to modern practice, is, in effect, only to throw ridicule on the study of their works; which, if taken as practical guides, would inevitably subject us to frequent indictments for *mala praxis,* to say nothing of occasional visits to the Old Bailey on charges of manslaughter. We do not deny that many valuable practical hints may be obtained from them; but we must repeat, that the chief use of ancient medical literature is to expand our views by throwing light on the history of the art and the progress of human knowledge. It is a study, of which it may in an especial manner be said, "Quamvis non faciat medicum, aptiorem tamen medicinæ reddit."

We now take leave of Mr. Adams, with the hope that the remaining part of his undertaking may be as successfully achieved as the first; and that his elaborate and useful work may obtain the place which it so justly merits in the library of every practitioner who is interested in the history and literature of his profession.

